# Acyclovir inhibition of IDO to decrease Tregs as a glioblastoma treatment adjunct

**DOI:** 10.1186/1742-2094-7-44

**Published:** 2010-08-06

**Authors:** Johan Söderlund, Sophie Erhardt, Richard E Kast

**Affiliations:** 1 Department of Physiology & Pharmacology, Karolinska Institutet, SE 171 77 Stockholm, Sweden; 2 Department of Psychiatry, University of Vermont, 22 Church Street, Burlington, VT 05401, USA

## Abstract

Regulatory T cells, Tregs, are a subset of lymphocytes that have immunosuppressive attributes. They are elevated in blood of glioblastoma patients and within this tumor's tissue itself. Indoleamine 2,3-dioxygenase, IDO, converts tryptophan to kynurenine. IDO activity enhances Treg formation by pathways that are unknown. Experimentally, inhibition of IDO decreases Treg function and number in rodents. The common anti-viral agent acyclovir inhibits IDO. Acyclovir may thereby decrease Treg function in glioblastoma. If it can be confirmed that Treg counts are elevated in glioblastoma patients' tumor tissue, and if we can document acyclovir's lowering of tissue Treg counts by a small trial of acyclovir in pre-operative glioblastoma patients, a trial of acyclovir effect on survival should be done given the current poor prognosis of glioblastoma and the well-established safety and low side effect burden of acyclovir.

## Background

Glioblastoma constitutes about half of all primary brain tumors and has a median survival time of 10 to 18 months with current standard treatment of maximal primary resection, irradiation, and temozolomide [1,2]. In the effort to find better treatments we reviewed past research on immunosuppressive lymphocytes in glioblastoma and looked for data that might indicate a clinically realizable path using currently available drugs to inhibit immunosuppressive lymphocytes.

An established antiviral drug, acyclovir, is known to inhibit indoleamine 2,3-dioxygenase (IDO) [3-5], an enzyme that is important in development of immunosuppressive lymphocytes. This paper outlines the experimental data generating this story and suggests that acyclovir might be a potential treatment adjunct for glioblastoma if past research can be confirmed.

### Tregs and the kynurenine pathway

Multiple lines of evidence point to a mild but clear state of systemic immunosuppression in patient with glioblastoma [6-9]. Why or how this comes about is not clear [6]. One probable contributor to this immunosuppressed state is IDO-mediated biasing of immune responses as outlined below.

Professional antigen presenting cells, such as dendritic cells (DCs), in addition to activating effects on cytotoxic T cells, may also recruit Foxp3-expressing CD4+CD25+ regulatory T cells (Tregs) to suppress cytotoxic responses. Trials of DC-based immunotherapy for glioblastoma [9,10] are currently being pursued, but these are hampered by the fact that DCs may have either an immunostimulatory or immunosuppressive phenotype [9,10]. As in the case for Tregs, the immunosuppressive DC phenotype is associated with IDO, as discussed below.

In vitro-generated Tregs express interleukin-2 (IL-2) receptors (CD25) but, unlike cytotoxic T cells, they do not proliferate or produce IL-2 upon ligation of T cell receptors (TCRs). In contrast, they inhibit IL-2 production by and TCR-induced proliferation of co-cultured T cells [11-13]. However, in vivo, Tregs themselves may well proliferate vigorously in response to TCR ligation while retaining their proliferation-inducing activity on effector lymphocytes [11-13]. This suggests that, in vivo, Tregs are active and specific participants in the suppression of antigen-driven immune responses.

Outside the thymus, de novo recruitment of Tregs is associated with tryptophan metabolism along the kynurenine pathway. The first step in the kynurenine pathway is conversion of tryptophan to formylkynurenine via the rate-limiting enzyme IDO or, in liver, via the related enzyme tryptophan 2, 3 dioxygenase (TDO)[14,15].

Activation of IDO in DCs during Treg recruitment is a well-replicated finding [16-21]. Diminished availability of tryptophan down-regulates the mammalian target of rapamycin, mTOR [22]. Diminished mTOR expression in DCs increases the recruitment and generation of FoxP3 expressing Treg [23,24]. Moreover, inhibition of IDO by 1-MT, an experimental IDO inhibitor, has been shown to inhibit Treg recruitment by plasmacytoid DCs while the addition of exogenous kynurenine enhances Treg recruitment by plasmacytoid DCs [25].

The determinants of generation of DC function to immunity or tolerance are unclear, but their environmental flexibility may be substantial [9]. Thus, the ligation of co-stimulatory molecule B7-1 on DCs by soluble or T cell membrane-bound cytotoxic T lymphocyte antigen-4 (CTLA-4) converts DCs to a tolerogenic phenotype [26] accompanied by evidence of increased IDO-dependent tryptophan catabolism [26]. This tolerogenic phenotype is obviated by enhanced proteosomal IDO degradation [26]. Immunogenic DCs lack IDO synthetic ability but acquire a tolerogenic phenotype if exposed to IDO-competent DCs and their paracrine functioning kynurenines [27].

CD8-DCs do not produce transforming growth factor-beta (TGF-β). Exogenous TGF-β will induce IDO in such cells and turns them from immunogenic into tolerogenic cells [28].

### Acyclovir

Introduced in the early 1980's, acyclovir was the fifth antiviral drug to see common use. It is thought to inhibit a specific thymidine kinase of certain Herpes viruses, most notably Herpes simplex [29]. Acyclovir is renally excreted with a circulating T1/2 of 3 hours. It is a well-tolerated, inexpensive drug with few side effects that is marketed worldwide [29].

Acyclovir has been shown to inhibit both IDO and TDO in homogenates of rat intestine [4] and liver [3]. Rat liver TDO is inhibited by acyclovir administered in vitro as well as in vivo [3]. The superoxide anion scavenging properties of acyclovir have been suggested as the mechanism underlying these actions [4,5], since superoxide anion is needed to activate the IDO/TDO enzyme.

Acyclovir is commonly used against Herpes labialis outbreaks, a condition often associated with or provoked by life stressors, presumably secondary to stress-related temporary loss of immune control. As discussed above, activation of the TDO/IDO complex is associated with recruitment of Tregs and thus suppression of specific immune responses. Since TDO is stimulated by cortisol and other adrenal corticosteroids [30] we suggest that one mechanism underlying stress-related Herpes labialis outbreaks is increased activity of TDO, which upregulates Treg activity. Stressors readily and dramatically increase adrenal synthesis of cortisol in all mammals that have been tested.

### Tregs in glioblastoma

T-cell proliferative defects are readily demonstrated in glioblastoma patients, and Treg overrepresentation is contributory to that state [31].

A core finding pointing to the potential utility of acyclovir use in glioblastoma is that glioblastoma cells are readily stimulated by interferon-γs and other stimuli, to upregulate IDO [32-36]. Although Tregs could be formed independently of IDO activation, the upregulation of IDO found within glioblastoma tissue is likely responsible, in part, for the enhanced presence of Tregs [37-41] within this tumor's tissue.

The concept and phenomenon of Treg-assisted evasion of specific immune responses against tumor antigens has been explored in other cancers. As examples: a) metastatic malignant melanoma expression of IDO is associated with increased numbers of Tregs, and higher numbers of such cells correlate with shorter survival [42], or b) the presence of Foxp3+ Tregs in breast cancer is associated with more advanced and aggressive disease [43].

The high number of FoxP3+ Treg cells in glioblastoma tissue [37-41], as compared to its complete absence in normal brain tissue, suggests that glioblastoma growth may benefit from the immunosuppressive activity of Tregs, and that glioblastoma patients will benefit from inhibiting Treg development with acyclovir. Although the degree of Treg infiltration does not have prognostic significance [38], experimental systemic Treg depletion has been shown to prolong survival in a murine orthotopic glioblastoma model, if depletion is done early after tumor implantation [39].

In addition to Tregs, myeloid cells with immature antigen-presenting phenotype are found within glioblastoma tissue [7,9]. Such cells can recruit circulating Tregs. In support of such a notion, monocytes co-cultured in vitro with glioblastoma cells acquire a phenotype characterized by high surface expression of TGF-β [7]. Increased production of TGF-β, elevated surface expression of TGF-β receptors, and activation of its signaling pathways has been shown to be important elements of glioblastoma growth promotion [8].

Freshly resected glioblastoma tissue synthesizes and secretes CCL2, a chemokine also associated with the recruitment of Treg. CCL2 is the only chemokine detected in glioblastoma tissue by enzyme-linked immunosorbent assay, indicating that CCL2 may be the principal chemokine for Treg migration to glioblastoma tissue [44]. CCL2 is a 13 kDa chemokine, also known as monocyte chemoattractant protein-1 [45].

A wealth of data implicates CCL5 as a paracrine/autocrine growth factor in glioblastoma [46]. The receptor for CCL5, CCR5, heterodimerizes with CCR2, the cognate receptor for CCL2. When the half of that dimer corresponding to CCR5 is blocked, CCL2 cannot signal even though binding to CCR2 is unimpaired. The recently approved and marketed anti-HIV drug maraviroc blocks CCL5 signaling at CCR5 [46]. Maraviroc has been proposed as treatment adjunct for glioblastoma based on documentation of CCR5 presence in glioblastomas and CCR5 function in glioblastoma growth stimulation [46]. Maraviroc therefore would have potential for inhibiting Treg migration to glioblastoma tissue as well as inhibition of CCR5 growth stimulation itself. Miraviroc may lower glioblastoma tissue Tregs in addition to acyclovir.

A second compelling reason to investigate acyclovir comes from the collective data of Cobbs et al. [47], Scheurer et al. [48], Mitchell et al. [49], and Prins et al. [50]. These researchers report detection of cytomegalovirus (CMV) in peripheral blood leukocytes and tumor tissue of glioblastoma patients but not in other conditions affecting the brain [47-50], indicating a potential role of CMV in glioblastoma pathogenesis.

Thus, acyclovir may prolong survival of glioblastoma patients also via its inhibition of thymidine kinase, which is expressed by CMV-infected cells. Another antiviral agent, gancyclovir, is marginally more efficient than acyclovir against CMV and may therefore also be interesting to test as adjunct to current glioblastoma protocol, although its inhibitory activity against IDO remains unknown.

Since reactivation of CMV either due to glioblastoma-associated immunosupression or secondary to temozolomide treatment is occasionally seen [51], acycloivir may be of service here too.

In accordance with our hypothesis of induction of IDO and recruitment of Tregs, a case study of a glioblastoma patient has described the development of a strong CMV-specific T cell response elicited by treatment with autologous tumor lysate-pulsed DCs [50].

## Conclusion

Given the short survival with current treatments following a diagnosis of glioblastoma and the low risks of acyclovir, a human trial of acyclovir as adjunctive treatment is warranted if preliminary trials in normal individuals show evidence of acyclovir suppression of circulating Tregs.

The hypothesis presented in this paper could be easily tested by quantitative immunocytophoresis of circulating Tregs in peripheral blood of normal volunteers before and after several days of acyclovir 200 mg p.o. four times daily. The safety of this dose is clear in that such treatment is commonly given without laboratory monitoring to patients to shorten an outbreak of Herpes simplex. Alternatively a naturalistic study of circulating Tregs by immunocytophoresis in people entering such Herpes outbreak treatment could give a good indication whether to proceed to a trial in glioblastoma or not.

## Competing interests

The authors declare that they have no competing interests.

## Authors' contributions

All authors contributed equally to all aspects of this work and have read and approved the final manuscript.
